# Elucidating the impact of low doses of nano-formulated benznidazole in acute experimental Chagas disease

**DOI:** 10.1371/journal.pntd.0006119

**Published:** 2017-12-21

**Authors:** Marcela S. Rial, María L. Scalise, Eva C. Arrúa, Mónica I. Esteva, Claudio J. Salomon, Laura E. Fichera

**Affiliations:** 1 Instituto Nacional de Parasitología “Dr. Mario Fatala Chaben”, ANLIS “Dr. Carlos G. Malbrán”, Ministerio de Salud, Buenos Aires, Argentina; 2 Area Técnica Farmacéutica, Departamento de Farmacia, Facultad de Ciencias Bioquímicas y Farmacéuticas, Universidad Nacional de Rosario, Rosario, Argentina; 3 Consejo Nacional de Investigaciones Científicas y Técnicas (CONICET), Buenos Aires, Argentina; University of Texas at El Paso, UNITED STATES

## Abstract

**Background:**

Chagas disease is a neglected parasitic infection caused by the protozoan *Trypanosoma cruzi (T*. *cruzi)* that affects more than 6 million people, mainly in Latin America. Benznidazole is still the drug of choice in many countries to treat it in spite of its dosage regimen and adverse side effects such as such as allergic dermatitis, peripheral neuropathy and anorexia. Thus, novel, safer, and more efficacious treatments for such neglected infection are urgently required.

**Methodology:**

In this study, the efficacy of orally administered low doses of benznidazole (BNZ) nanoparticles was evaluated during the acute phase in mice infected with *T*. *cruzi* Nicaragua (*Tc*N) that were immunosuppressed during the chronic stage of the disease. Moreover, the production of *T*. *cruzi*-specific antibodies, cardiac tissue inflammation and reactive oxygen species generation by Vero cells treated with both BNZ nanoparticles (BNZ-nps) and raw BNZ (R-BNZ) were also evaluated.

**Principal findings:**

*T*. *cruzi* infected mice treated with 10, 25 or 50 mg/kg/day of BNZ-nps survived until euthanasia (92 days post infection (dpi)), while only 15% of infected untreated mice survived until the end of the experiment. PCR analysis of blood samples taken after induction of immunosuppression showed that a dosage of 25 mg/kg/day rendered 40% of the mice PCR-negative. The histological analysis of heart tissue showed a significant decrease in inflammation after treatments with 25 and 50 mg/kg/day, while a similar inflammatory damage was observed in both infected mice treated with R-BNZ (50 mg/kg/day) and untreated mice. In addition, only BNZ-nps treated mice led to lower levels of *T*. *cruzi*-specific antibodies to 50–100%. Finally, mammalian Vero cells treated with BNZ-nps or R-BNZ lead to a significant increase in ROS production.

**Conclusions:**

Based on these findings, this research highlights the *in-vitro/in-vivo* efficacy of nanoformulated BNZ against *T*. *cruzi* acute infections in immunosuppressed and non-immunosuppressed mice and provides further evidence for the optimization of dosage regimens to treat Chagas disease.

## Author summary

Chagas disease is a neglected parasitic infection caused by the protozoan *Trypanosoma cruzi (T*. *cruzi)* that affects more than 6 million people, mainly in Latin America. Benznidazole is still the drug of choice in many countries to treat it in spite of its dosage regimen and adverse side effects such as such as allergic dermatitis, peripheral neuropathy and anorexia. In this study, the efficacy of low doses of benznidazole, formulated as nanoparticles, against *T*. *cruzi* acute infections in immunosuppressed and non-immunosuppressed mice was investigated in order to establish future treatment strategies. *In-vivo* experiments showed that all infected mice treated with low doses of nanoformulated benznidazole survived until the end of the assay (92 dpi), while only 15% of infected untreated mice survived to the end of the same period of time. Moreover, such novel formulation was able to decrease the parasite burden and, consequently, heart inflammation and lesions were significantly reduced. Clearly, low doses of benznidazole exhibited, at least, the same efficacy in infected mice as the usual dose, confirming the usefulness of nanoformulated benznidazole for an improved treatment of Chagas disease.

## Introduction

Chagas disease, a neglected disease caused by the protozoan *T*. *cruzi* and transmitted by triatomine bugs, is the most prevalent parasitic disease in Latin America. It affects more than 6 million people causing, approximately, 12000 deaths annually and nearly 100 million people are at risk of acquiring this infection. [1, 2]. In the last two decades, Chagas disease has also been detected in other regions including Canada, Japan and Europe. It should be noted that this parasitic infection is an emerging disease in many regions of North America, as recently reported by García et al. [3]. This infection proceeds in two different clinical phases: an acute stage and a chronic stage. The acute phase lasts for 45–60 days and symptoms are generally absent or mild. During the chronic phase up to 30% of patients present cardiac failures including arrhythmias, cardiomyopathy and thromboembolism. Other manifestations may be emphysema, stroke, megaesophagus, gastric ulcers, and megacolon [4].

Benznidazole (BNZ), discovered more than 40 years ago, is one of the effective and therapies available to treat this neglected infection [5]. Even though it is widely prescribed, there are major concerns related with the frequency of serious side effects, including allergic dermatitis, gastrointestinal intolerance, anorexia, weight loss and sleeping disorders [6]. Several authors have reported different treatments in experimental murine model using lower doses of BNZ alone or combined with other drugs, to improve treatment efficacy and decrease adverse side effects [7, 8, 9]. Despite the fact that these reports have shown certain benefits in terms of survival rate and lower parasitemia, there is an urgent need to develop novel therapeutic alternatives using low doses of BNZ for further successful clinical translation. In this regard, nanoparticulate based drug delivery systems are an attractive and effective tool to overcome several drawbacks of conventional drug formulations, such as low solubility in biological fluids, erratic biopharmaceutical performance, and systemic drug toxicity [10]. However, only few attempts have been made to provide new solutions for the treatment of Chagas disease through nanotechnology platforms [11]. Recently, we have evaluated the effectiveness of BNZ nanoparticles (BNZ-nps) against *T*. *cruzi* trypomastigotes and against intracellular infection in mammalian cells and primary cardiac myocyte cells. BNZ-nps were evaluated against acute *T*. *cruzi* Nicaragua (*Tc*N) infection in mice, demonstrating that mice treated with BNZ-nps (10, 25 and 50 mg/kg/day) for 30 days and with BNZ-nps (50 and 25 mg/kg/day) for 15 days presented a 100% survival at 50 days post-infection (p.i.), while the animals treated with 10 mg/kg/day of BNZ-nps for 15 days showed a 70% survival rate [12]. In this study, we evaluated the efficacy of low doses of BNZ-nps administered during the acute phase in *Tc*N mice infected that were immunosuppressed during the chronic stage of the disease. Additionally, production of *T*. *cruzi*-specific antibodies, cardiac tissue inflammation and ROS production by Vero cells treated with this BNZ nanoformulations were also investigated.

## Materials and methods

BNZ (Abarax, lot 9978 A; Laboratorios Elea, Buenos Aires, Argentina) was provided by Instituto Nacional de Parasitología, ANLIS Malbrán, Ministerio de Salud de la Nación, (Buenos Aires, Argentina). Poloxamer 188 (P188) was donated by BASF SE (Ludwigshafen, Germany). Fetal bovine serum (FBS) was purchased from Gibco (Rockville, MD, USA). Horse serum was obtained from Internegocios SA (Córdoba, Argentina). Phorbol-12-Myristate-13-Acetate (PMA) was purchased from Sigma Chemical Co. (St Louis, MO, USA). Vero cells were obtained from ABAC (Pergamino, Argentina). All the other reagents and chemicals used for analytical purpose were of chromatography grade.

### Preparation of BNZ-nps

BNZ-nps were prepared by solvent diffusion method. Ethanol and water were used as solvent and antisolvent respectively at a ratio of 1:2. Briefly, BNZ (200 mg) was dissolved in ethanol (10 mL). Then, the organic solution was slowly injected dropwise (syringe 5 mL; needle 26 G) at a rate of 1 mL min^−1^ into water (20 mL) containing P188 (300 mg). Such dispersion was kept under magnetic agitation of 1000 rpm for 60 min. Then, the resulting nanodispersion was magnetically stirred (500 rpm) for 18 h at room temperature to allow solvent evaporation. Nanoparticles were then recovered by centrifugation for 20 min (15000 rpm), washed twice with distilled water and frozen overnight at -20 ^o^C [12].

### Lyophilization of BNZ nanosuspension

BNZ nanosuspensions were frozen and lyophilized using a Labconco FreeZone 4.5 L (Labconco, Kansas City, MO, USA) for 48 h at -40°C. The freeze-dried samples were diluted to original volume with distilled water. The calculation of the percentage of nanoparticle recovery (NR %) was performed in triplicate (Eq 1).

NR(%)=[mgBNZ-nps/(mgBNZ+mgP188)]x100 (1)

where *mg BNZ-nps* is the weight of the recovered nanoparticles, *mg BNZ* and *mg P-188* are the initial weight of the BNZ and the poloxamer, respectively.

NR (%) refers to the amount (%) of nanoparticles obtained after the freeze-drying process.

### Particle size determination

BNZ-nps particle size was determined by experiments of dynamic light scattering at a scattering angle of 90 to 25°C using a Nanoparticle Analyzer SZ-100. The parameters measured were polydispersity index (PDI) and z-average diameter. Zeta potential (ζ) was measured using a two-face cuvette using the same equipment. Nanoparticle solutions were prepared in a solution of Tween 20 (0.1% w/v), previously filtered. The sample contained no more than 0.01% by volume of particles. The measurements were performed in triplicate and the data obtained was expressed as mean±SD.

### Saturation solubility studies

The saturated solubility of BNZ-nps samples was assayed by adding an excess amount (100 mg) of each sample in a vial with 5 mL of solution of medium (distilled water, pH 6.3). For comparison purposes, R-BNZ and the corresponding physical mixture formed by BNZ and P188 (1:1.5 ratio) were as well evaluated under the same conditions. The samples were shaken in a Boeco orbital shaker (Hamburg, Germany) at 25 ^o^C and 150 rpm until equilibrium was reached (72 h). Upon equilibrium the samples were filtered through a 0.45 μm filter and measured by UV at 324 nm. All experiments were carried out in triplicate.

### Parasites

*Tc*N was obtained from the intestinal content of a *Triatoma dimidiata* vector captured in an urban endemic area of Nicaragua. The isolate was characterized in our laboratory [9]. Trypomastigotes were obtained from cell cultures using kidney epithelial cells of the African green monkey, VERO cells (ABAC, Pergamino, Argentina).

### Animal model

**Fig 1:**
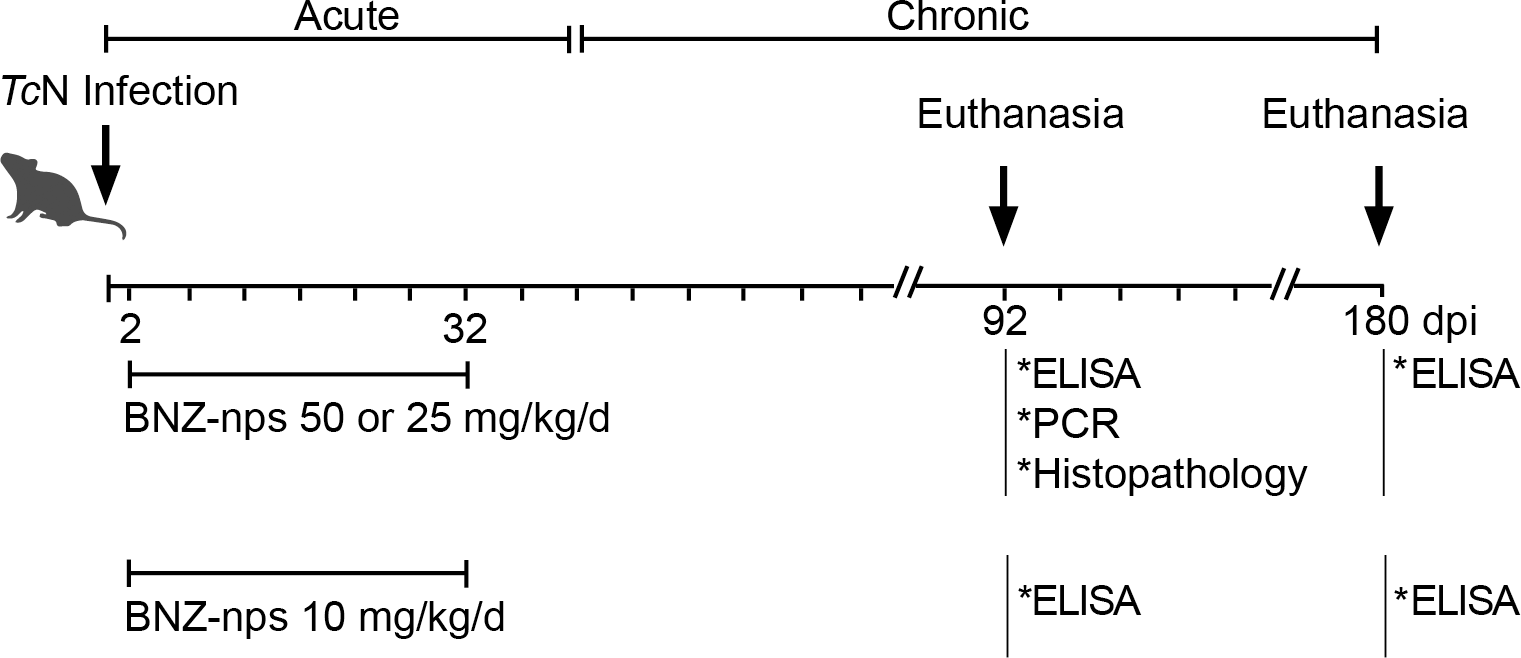
Schedule of treatment with BNZ-nps 50, 25 and 10 mg/kg/day.

Five groups of ten one-month-old female C3H/HeN mice were inoculated intraperitoneally with 1000 culture-derived trypomastigotes of the *Tc*N isolate. 1000 trypomastigotes/mouse correspond approximately to 100 times of the LD50. Infected mice were divided into the following groups (n = 10): (1) infected mice without treatment, (2) infected mice treated with raw BNZ with daily doses of 50 mg/kg body weight for 30 days (2 to 32 dpi) (R-BNZ 50), (3) infected mice treated with BNZ nanoparticles for 30 days with daily doses of 50 mg/kg/day (BNZ-nps 50), (4) infected mice treated with BNZ nanoparticles for 30 days with daily doses of 25 mg/kg/day (BNZ-nps 25), (5) infected mice treated with BNZ nanoparticles for 30 days with daily doses of 10 mg/kg/day (BNZ-nps 10). R-BNZ and BNZ-nps were dispersed in olive oil and administered to mice through oral gavage. The control group received a mock-treatment with olive oil alone. The course of infection was assessed by monitoring parasitaemia and survival rates. Parasitaemia (n = 5 mice per day) was scored as previously described [13]. The area beneath the parasitaemia curves was determined using Graph Prism 5.0. Mice survival rates were checked daily. Infected mice treated in the acute phase and untreated animals were euthanized after 3 months of follow-up (Fig 1).

### Induction of immunosuppression

**Fig 2:**
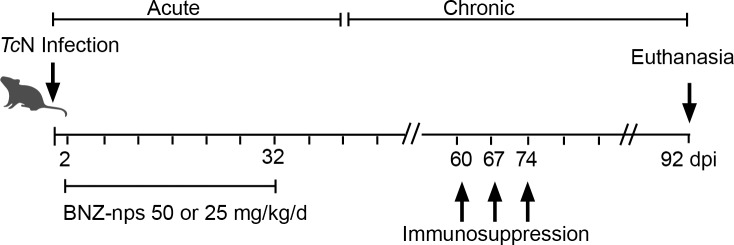
Schedule of immunosuppression with cyclophosphamide treatment.

After 60 days of inoculation, the animals were immunosuppressed with cyclophosphamide (Microsules Argentina, Garín, Buenos Aires, Argentina). The immunosuppression protocol consisted of three cycles of 50 mg of cyclophosphamide/kg of body weight, for four consecutive days, with an interval of 3 days between each cycle (Fig 2) [14]. After the last cycle of cyclophosphamide treatment, parasitemia was evaluated in fresh blood collected from the mouse’s tail for 10 days and the number of parasites was estimated as described by Brener [13] and by PCR.

### Detection of *T*. *cruzi* in peripheral blood by PCR

Peripheral blood from infected treated and untreated mice was obtained by retro-orbital venipuncture after completing the drug treatments at 3 months post-infection (n = 5 samples per treatment). The samples were mixed with an equal volume of guanidine-HCl 6 M, EDTA 0.1 M, pH 8, kept at room temperature for 1 week and then at 4°C until use. The PCR was performed with primers 121 and 122 (121 AAATAATGTACGGG(T/G)GAGATGCATGA, 122 GGTTCGATTGGGGTTGGGTAATATA), which amplify a 330-bp sequence from kinetoplast DNA (k). The amplification reactions were achieved in a volume of 25 μl, consisting of 2.5 μl of the 10× Taq Platinum buffer (100 mM Tris–HCl, pH 8.3; 500 mM KCl), 2.5 μl of a dNTP mixture (200 M of each), 2.5 μl of each primer, 0.2 µl of Taq Platinum (Life Technologies, NY, USA), 1.75 μl of MgCl_2_ solution and 5 μl (approximately 100 ng) of DNA per sample. Gel electrophoresis was performed using 2% agarose and TAE buffer in the presence of 0.5 g/mL of ethidium bromide. The detection limit was 0.002 parasites per assay, equivalent to 2 parasites/5 mL [15].

### Assessment of humoral immune responses specific for *T*. *cruzi*

Blood from uninfected and both infected treated and untreated mice was collected from the orbital venous sinus (500 μl) at 3 and 6 months post-infection. Serum samples were analyzed for IgG antibody levels by use of an Enzyme Linked Immunosorbent Assay (ELISA). A lysate preparation derived from epimastigotes of the *T*. *cruzi* Tulahuen strain, Tul_2_ stock (20 μg/mL) was used as source of antigen. Briefly, flat-bottomed (96 well) plates were coated overnight at 4°C with 50 μl/well of antigen diluted in carbonate buffer pH = 9.6. Plates were blocked for 1 h at RT with 100 μl/well of 5% skimmed milk in PBS. After being washed 3 times with PBS-0.05% Tween_20_ (PBS-T) plates were incubated with sera samples (1:50–1:400 dilution, 50 μl/well) for 30 minutes at 37°C. After washing with PBS-T, 50 μl/well of horseradish peroxidase-labelled goat anti-mouse IgG (Jackson) were added for 30 minutes at RT. The reaction was developed with 50 μl/well of *o*-phenylenediamine dihydrochloride, and stopped with 2N sulfuric acid. Optical density was read at 490 nm with an ELISA microplate reader (Dynatech). A cut-off value for significant decreases in antibody levels was set up as the mean minus two standard deviations of optical density, obtained from the sera of infected untreated control mice.

### Histopathological studies

Heart tissues were removed from treated and infected untreated mice, fixed in 10% formaldehyde solution and embedded in paraffin. Five-micron tissue sections were stained with haematoxylin-eosin (H&E) stain and evaluated by light microscopy. Tissues from 8 different areas of the heart (left and right atria, upper and lower halves of each ventricular wall and septum) were scored according to the extension of inflammation.Each section was assigned (0) = absent/none, (1) = focal or mild myocarditis with 1 foci, (2) = moderate with 2 inflammatory foci, (3) = extensive with generalized coalescing of inflammatory foci or disseminated inflammation with minimal necrosis and retention of tissue integrity, and (4) = severe with diffused inflammation, interstitial edema, and loss of tissue integrity, as already reported [16]. An average of the values found in the eight sections of the heart represents an estimate of the degree of cardiac tissue inflammation.

### Quantification of reactive oxygen species (ROS) in Vero cells

The production of oxygen species (ROS) was quantified by the fluorescence assay based on 2`,7`-dichlorofluorescein-diacetate (H2DCFDA) [17]. Briefly, 1x10^4^ Vero cells were seeded per well, in 96-well plates, that were incubated with RPMI plus 5% SFB overnight at 37°C, in a humidified atmosphere with 5% CO_2_. They were then washed to remove traces of serum from the cultures and incubated for 30 minutes at 37°C, with 10 μM H2DCFDA (Life Technologies, NY, USA). Then, the different treatments were added to the cell cultures: RPMI, PMA, R-BNZ and BNZ-nps to determine the production of intracellular ROS at 15, 30 and 60 min. Each experiment was performed in triplicate, with three repetitions. Fluorescence was measured on a microplate reader, Glomax-Multi + detection system (Promega, Wisconsin, USA).

### Statistical analysis

The survival curve was determined using Graph Prism 5.0. The statistical significance of data (*p* < 0.05) were analyzed by the Student’s t-test and the morphometric results of the different treatment protocols were compared by analysis of variance (ANOVA) test. The comparison of the treatments analyzed by PCR were performed by Fisher's test.

### Ethics statement

All procedures involving animal use followed the rules of the ethical legislation and regulatory entities established in Argentina and were approved by the Bioethics Committee of the National Institute of Parasitology “Dr. Mario Fatala Chaben” (Register RENIS Nº: 000028), and met the international recommendations for the use of laboratory animals (World Medical Association in the Declaration of Helsinki).

## Results

### BNZ nanoparticles

The preparation of the BNZ-nps using P188 as a stabilizer was carried out according to our previous work [12]. Following the nanoprecipitation methodology, BNZ was dissolved in ethanol (solvent phase) and added to an antisolvent phase (aqueous phase) containing a stabilizer molecule (P188). BNZ-nps physicochemical characterization, by means of dynamic light scattering experiments indicated a mean particle size of 63.3 nm, a zeta potential of -18.30 mV and a size distribution (PdI) of 3.35 nm. The obtained values of the particle size indicated a high reproducibility (less than 3% of deviation between triplicates) while the obtained polydispersity indexes suggest the formation of a homogeneous BNZ nanosuspension. On the other hand, the recovery of the BNZ-nps (98%) after the freeze-drying process indicated that the both the process and the stabilizer were adequately chosen. The saturation solubility of BNZ-nps was found to be 3.99 mg/mL, while the saturation solubility of R-BNZ and BNZ-P188 mixture were 0.4 and 0.7 mg/mL, respectively. The characterization of the BNZ-nps are described in Table 1.

**Table 1:** Physicochemical characterization of BZN-nps. All measurements were carried out in triplicate.

	Particle size (nm)	Zeta potential (mV)	PdI (nm)	BNZ-nps (%)	Solubility (mg/mL)
BNZ-nps	63.30 ± 2.82	-18.30±1.00	3.35±0.10	98.00±0.10	3.75±0.12

### Course of infection

**Fig 3:**
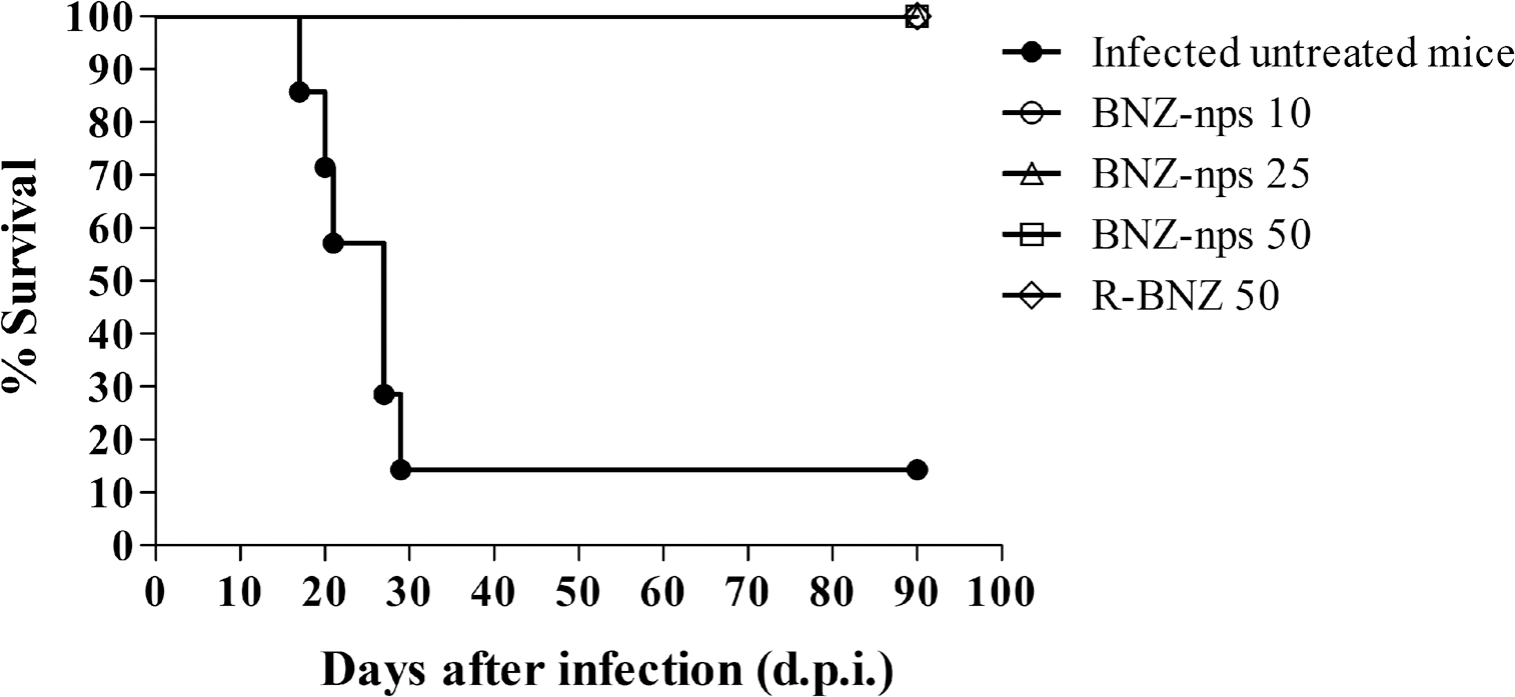
Survival curve in C3H/HeN mice infected with 1000 trypomastigotes of *Tc*N and treated with 30 oral doses of R-BNZ 50, BNZ-nps 50, 25 and 10 mg/kg/day, respectively. To assess differences among survival curves, a log-rank test of Kaplan–Meier was performed, which showed significant differences between both infected treated and untreated mice (p < 0.0001).

In order to determine whether BNZ-np treatments affect the course of infection and survival rate of C3H/HeN mice infected intraperitoneally with trypomastigotes of the *Tc*N isolate, drug formulation were orally applied at 10, 25 and 50 mg/kg/day and compared with R-BNZ at a dose of 50 mg/kg/day. Control mice remained untreated and were also infected. Oral treatment was started 2 dpi and given daily for 30 consecutive days. As seen in Fig 3, all infected mice treated with both R-BNZ and BNZ-nps survived until the end of the experiment (92 dpi). In the group treated with olive oil without BNZ, only 15% of the mice survived. At 92 dpi, all surviving mice were euthanized.

### Humoral immune responses specific for *T*. *cruzi* following chemotherapy

**Fig 4:**
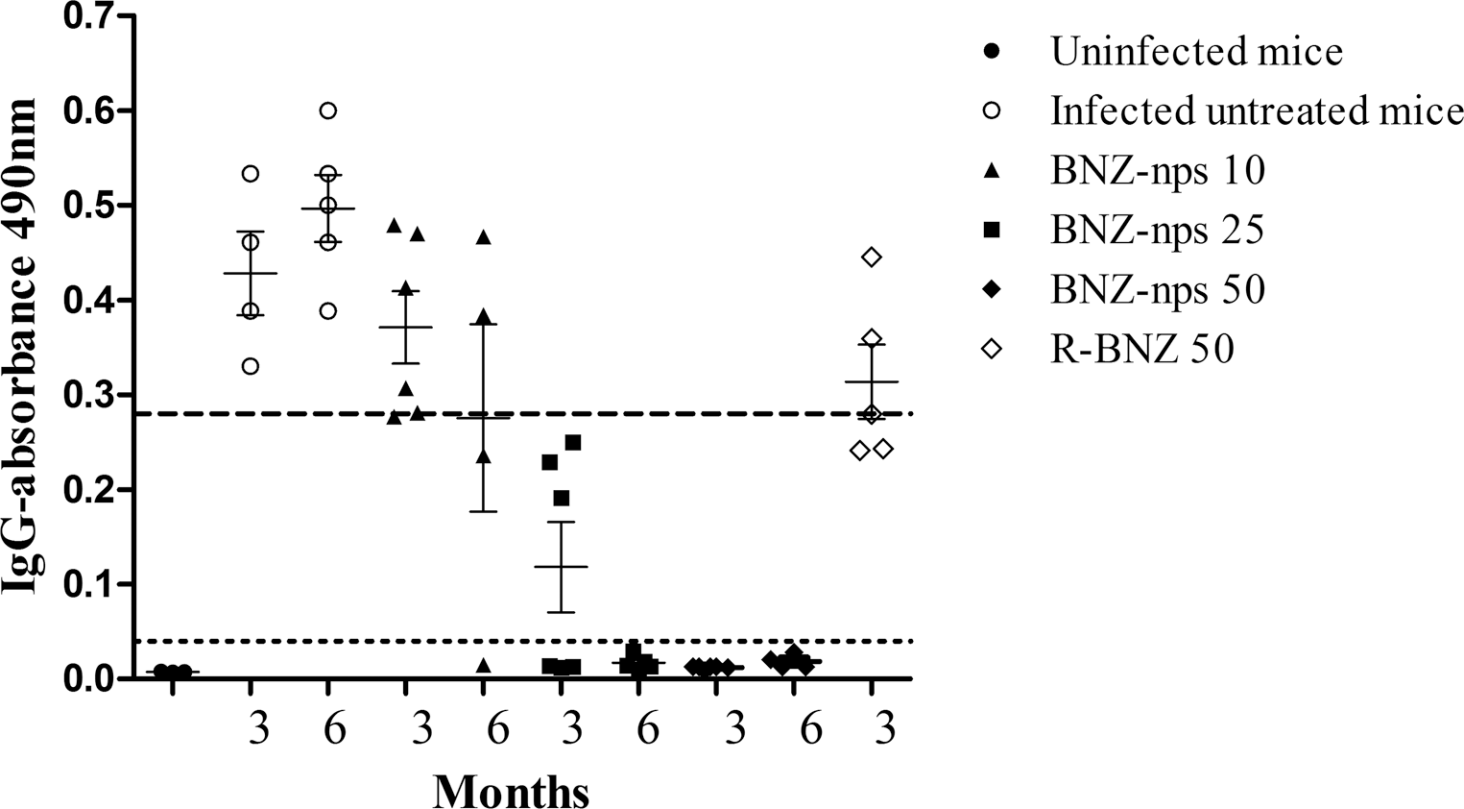
IgG levels specific for an epimastigote lysate preparation measured at 3 and 6 months dpi. Serum samples from *TcN*-infected untreated and treated mice with R-BNZ 50 mg/ kg/day, BNZ-nps 10, 25 and 50 mg/kg/day, respectively were analysed for IgG antibody levels specific for *T*. *cruzi* antigens by ELISA. Each dot represents antibody levels from individual mice. Horizontal lines show median values. The cut-off value of antibody levels were described in the Materials and Methods section and are represented by the horizontal dotted lines. All comparisons were performed with infected untreated mice at the same time, **p<0.001, *** p<0.0001.

The immune responses specific for *T*. *cruzi* were assessed in all experimental groups (Fig 4). All mice treated with BNZ-nps at 25 mg/kg/day showed a decrease in *T*. *cruzi*-specific antibodies compared to titers of infected control mice. In particular, no antibodies could be detected in 50% of the animals at 3 months and 100% at 6 months. When BNZ-nps was applied at 50 mg/kg/day, all mice displayed negative titers in *T*. *cruzi*-specific antibodies at 3 months post-infection. No differences in antibody titer were observed among mice receiving R-BNZ 50 mg/kg/day and untreated controls, as described by our group [18].

### Detection of *T*. *cruzi* DNA in mouse blood

**Fig 5:**
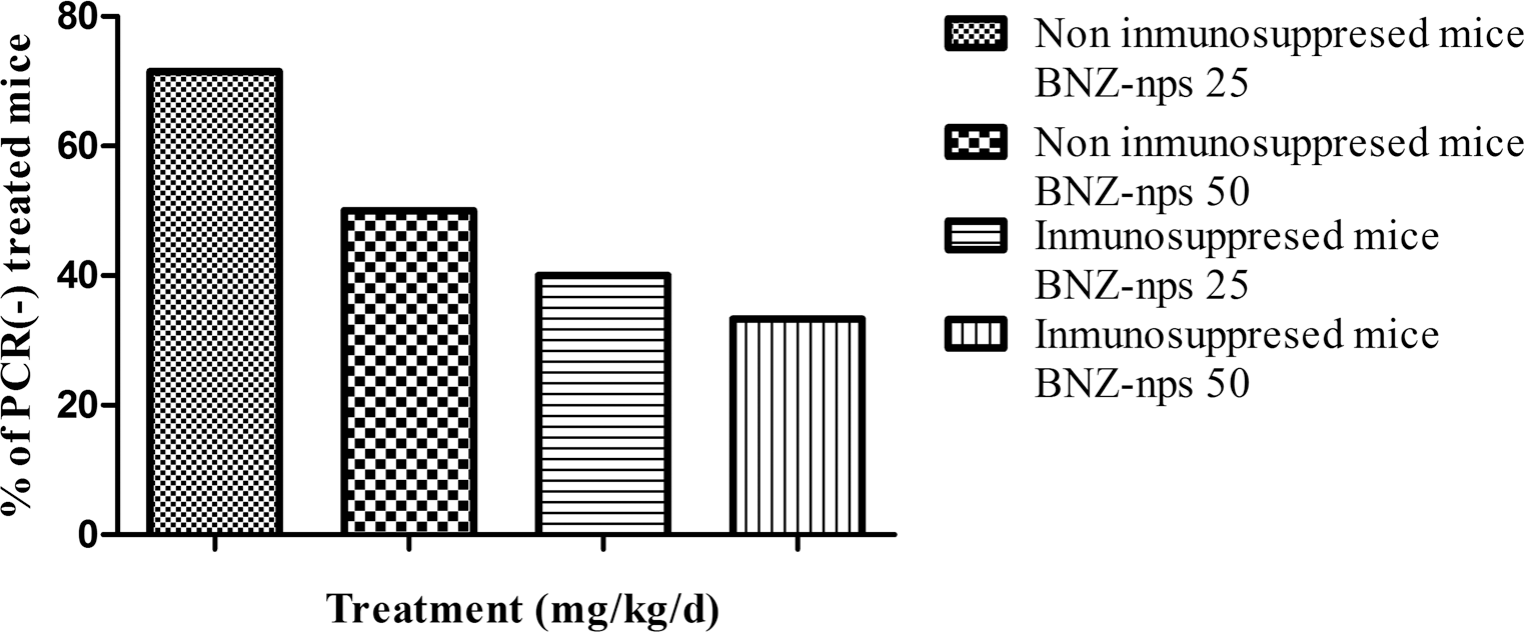
Percentage of negative samples by PCR. Mouse blood samples obtained from immunosuppressed (n = 11) and non-immunosuppressed (n = 13) TcN-infected mice treated with BNZ-nps at 25 mg/kg/day or 50 mg/kg/day were analyzed by PCR amplification of a satellite DNA. No differences were observed between BNZ-nps doses by Fisher exact test p = 1.0.

As peripheral blood parasites were not found by optical microscopy in mice treated with BNZ-nps, the efficiency of these new formulations was assessed by immunosuppression. Thus, the effectiveness of BNZ-nps against *T*. *cruzi* in *TcN*-infected mice was investigated by PCR of mouse blood samples obtained from immunosuppressed and non-immunosuppressed mice. The immunosuppressed mice treated with BNZ-nps at 25 mg/kg/day and 50 mg/kg/day exhibited 40 and 33% of PCR negative samples, respectively. In the case of non-immunosuppressed mice, the percentage of infected mice decreased to 71% and 50% following the administration of 25 mg/kg/day and 50 mg/kg/day of BNZ-nps, respectively (Fig 5). In contrast, parasites were detected in blood from mice treated with R-BNZ 50, as already described [18].

### Histopathology

**Fig 6:**
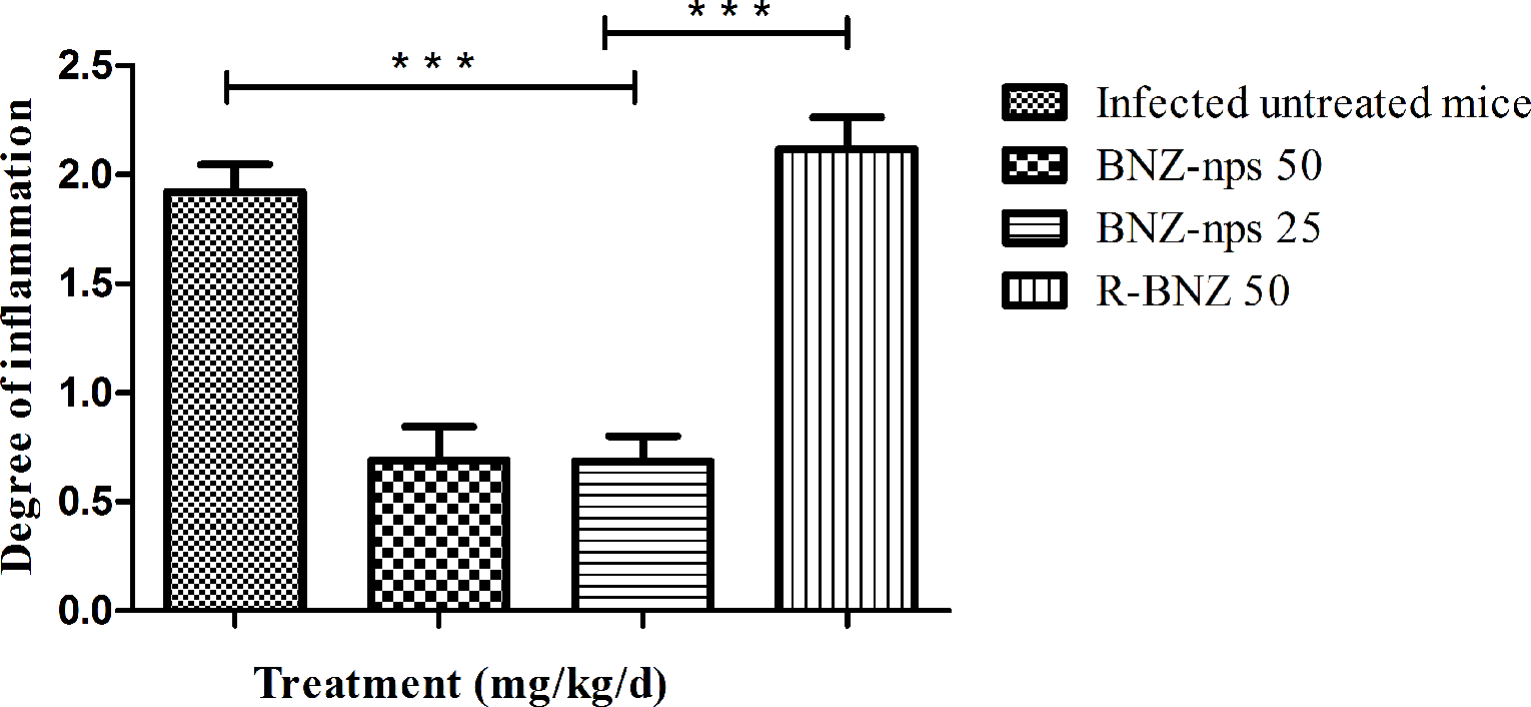
Evaluation of inflammation in mice infected with *Tc*N isolate and treated with BNZ-nps and R-BNZ as described in Fig 1. Data represent the morphometric quantification of inflammatory cells in heart tissue stained with haematoxylin-eosin (H&E). (***) p <0.0001 represents infected untreated and infected R-BNZ treated mice vs treated with BNZ-nps.

One of the most typical clinical manifestations of Chagas disease is related with cardiac tissue, which leads to the appearance of several heart pathologies including myocarditis and pericarditis [19]. In this study, we evaluated whether nanoformulated BNZ would have an impact over the cardiac tissue inflammation produced by *Tc*N strain. As seen in Fig 6, infected mice treated with R-BNZ (50 mg/kg/day) exhibited similar inflammatory damage to that of the infected untreated group. In contrast, a significant decrease of inflammatory cells in the heart tissue was observed after treatment with BNZ-nps at a dosage of 25 and 50 mg/kg/day.

**Fig 7:**
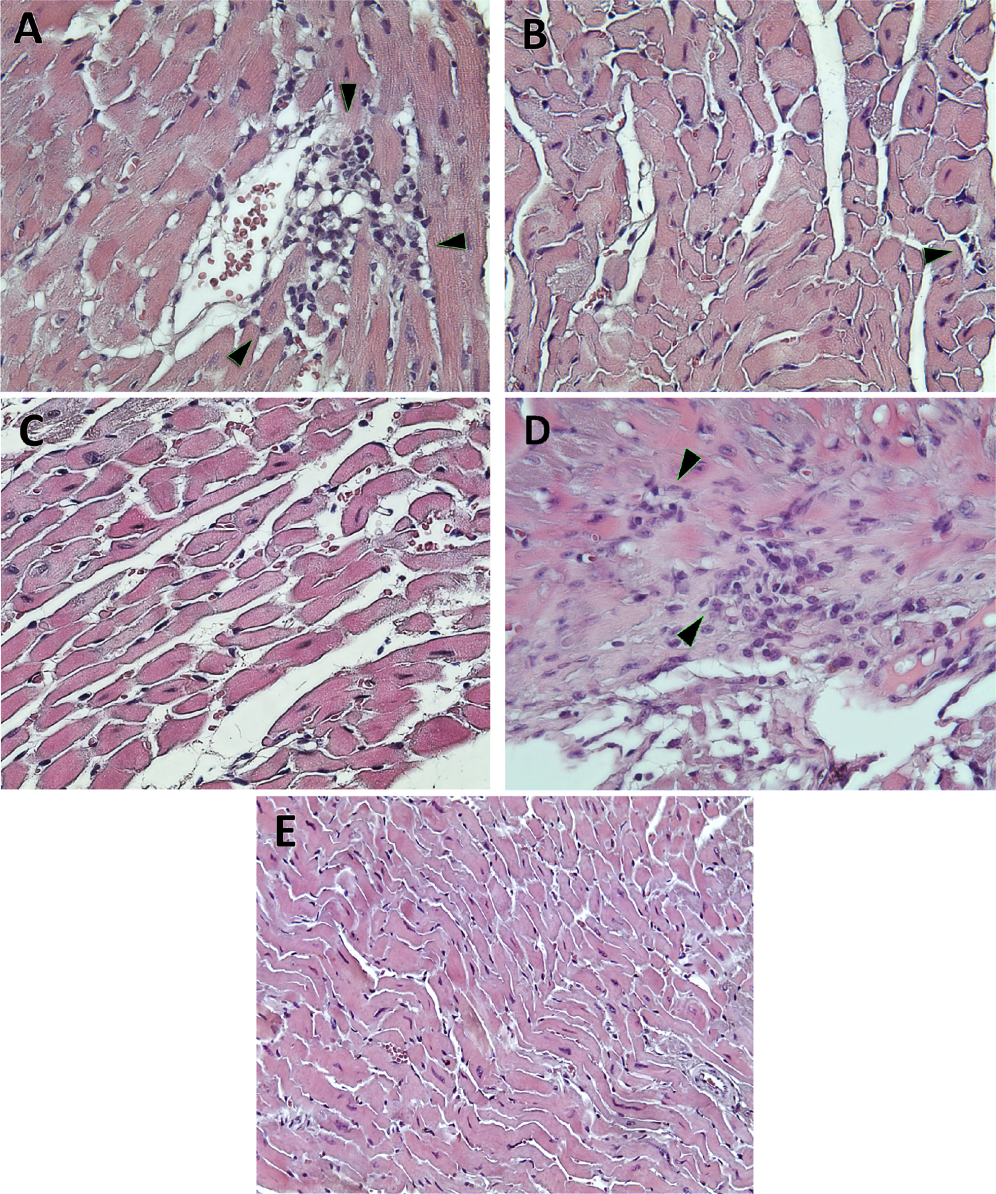
Heart tissue in chronic phase subjected to haematoxylin and eosin (H&E) staining. (A) Ventricle from untreated infected mice; (B) ventricle from treated infected mice with BNZ-nps 25 mg/kg/day; (C) ventricle from infected mice treated with BNZ-nps 50 mg/kg/day; (D) right auricle from treated infected mice with R-BNZ 50 mg/kg/day; (E) ventricle of uninfected mice. Magnification: 40X.

As shown in Fig 7A and indicated with an arrow, the myocardium of infected untreated mice showed extensive and multiple inflammatory foci of mononuclear cell infiltrates and some necrotic areas with structural alterations and fibrotic foci. On the other hand, parasite nests were absent in heart tissues of mice that survived the acute stage, as observed in Fig 7B and 7C. Cardiac tissue from infected mice treated with BNZ-nps 25 (7B) and BNZ-nps 50 mg/ kg/day (7C) did not show any damage or inflammation compared with the infected untreated animals (7A), while mice treated with R-BNZ 50 showed multiple inflammatory foci, as indicated by the arrows (7D). On the other hand, the tissue of uninfected mice is shown in Fig 7E.

### *In-vitro* ROS production

**Fig 8:**
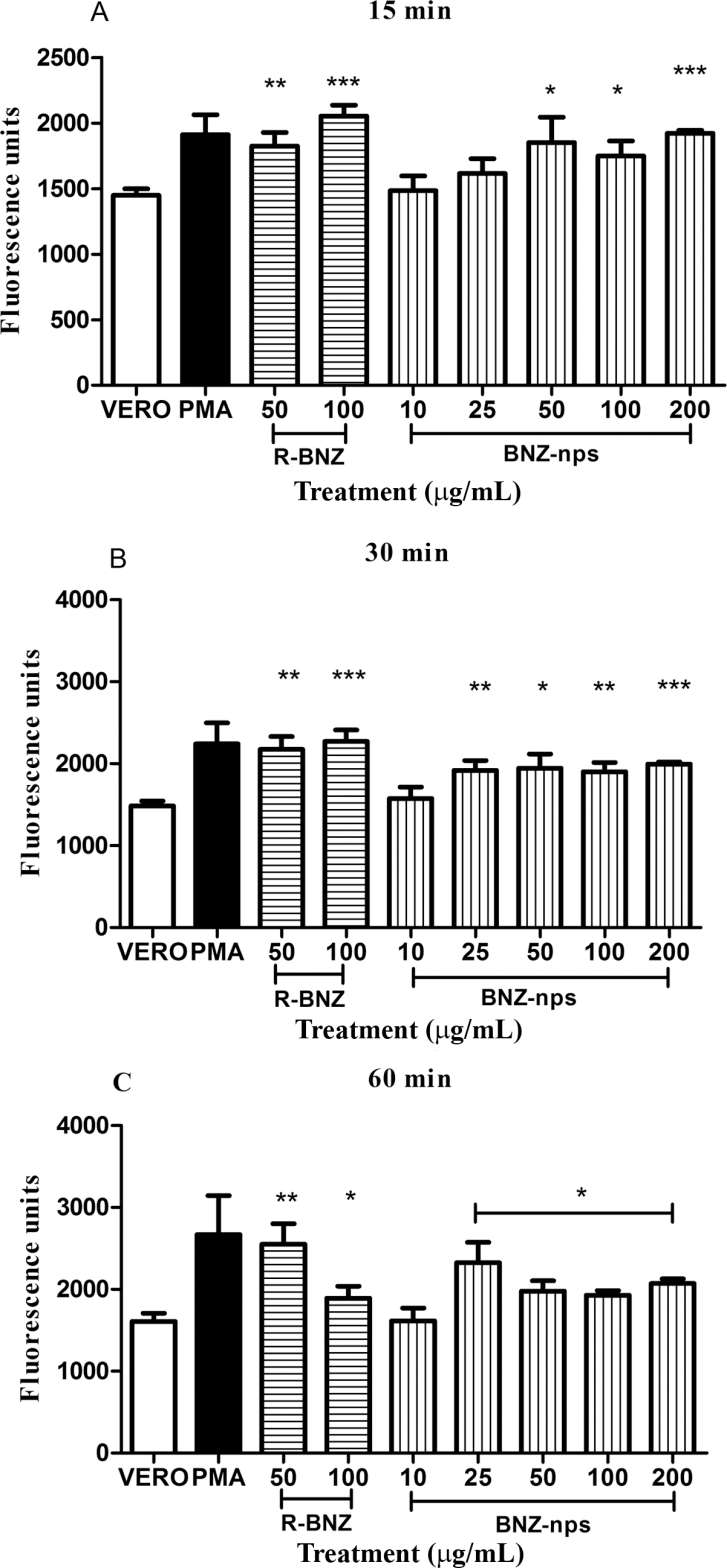
Reactive oxygen species (ROS) induction by BNZ-nps in Vero cells. Vero cells were pre-incubated with the fluorescent H2DCFDA probe and exposed to increasing concentration of R -BNZ (50 and 100 μg/mL) or BNZ-nps (10, 25, 50, 100 and 200 μg/mL). Fluorescence was evaluated at 15 min (A), 30 min (B) and 60 min (C) of incubation. PMA was used as a positive control. *p <0.05, **p <0.001 and ***p <0.0001 respect to the untreated control.

In order to evaluate whether BNZ-nps are able to induce ROS production, an *in-vitro* assay in Vero cells was performed. Such cells were incubated in the presence of different concentrations of BNZ-nps, or R-BNZ as control, for 15, 30 and 60 min. After 15 min incubation, an increase in ROS production, measured by fluorometry using the H2DCFDA probe was observed by means of 50 μg/ml BNZ-nps (Fig 8A). However, after 30 (Fig 8B) and 60 min (Fig 8C) of incubation, ROS production was detected with a lower BNZ-nps concentration (25 μg/ml). On the other hand, R-BNZ 50 and 100 ug/ml induced ROS at any time tested.

## Discussion

Here we report for the first time the impact of a dose-response treatment with BNZ nanoparticles on acute experimental Chagas disease. Even though BNZ is still the drug of choice for Chagas disease, its bioavailability is limited because of its low aqueous solubility and dissolution rate. Thus, reduction of particle size to nanoscale is a promising strategy to improve the biopharmaceutical performance of BNZ [20]. In 2002, BNZ multilamellar liposomes were developed in order to improve its absorption and bioavailability. The lipid carrier was prepared with a mixture of hydrogenated phosphatidylcholine, distearoyl-phosphatidylglycerol, and cholesterol [21]. Later, the same research group evaluated BNZ biodistribution and pharmacokinetic profile after parenteral administration of BNZ liposomes in healthy rats [22]. An increase in hepatic BNZ uptake was observed, but no effect on parasitemia levels in mice infected with *T*. *cruzi* RA strain was detected [22]. In the present work, the nanoprecipitation method, a widely used process for the formulation of nanocrystals and nanosuspensions, was selected for the preparation of BNZ-nps. This methodology presents several advantages including the need for few excipients (hydrophilic polymers and/or surfactants as stabilizers) and for pharmaceutical approved organic solvents. In this regard, ethanol (ICH class 3 solvent), comparatively less toxic than methanol, dichloromethane and chloroform (ICH class 2 solvents), was selected as solubilizing agent for BNZ [23]. Although nanoprecipitation by solvent diffusion is a convenient methodology for the preparation of nanoparticles, high free-energy surfaces may be originated during the process leading to a fast nanoparticle agglomeration phenomena. To avoid this, a linear polyoxyethylene-polyoxypropylene block copolymer consisting of a hydrophobic central segment of PPO and two hydrophilic side segments of PEO (P188) was selected as stabilizer. Due to its amphiphilic properties, the PEO units may surround the BNZ crystals surface causing the steric hindrance and avoiding, as a consequence, the agglomeration of the newly formed nanoparticles. On the other hand, the PPO unit leads to the adsorption on the BNZ particles surface [24]. As seen in this work, P-188 led to the formation of BNZ nanoparticles with mean particle size of 63.3 nm. It was also noted that the zeta potential was -18.30 mV, which is high enough for efficient stabilization, as described by Müller et al. [25]. As observed in Table 1, the saturation solubility assay of BNZ-nps was nearly 4.00 mg/mL, more than 10 times higher than R-BNZ and more than 5 times higher than the BNZ-P188 mixture. It is well known that the solubility of a molecule depends on the size of the particle and, therefore, reducing the size from the micrometer to nanometer scale would lead to a higher solubility. Moreover, the concentration of the stabilizer agent (P188) adsorbed onto the particle surface would have a direct impact on the solubility of BNZ. As shown herein for the first time, the nanoprecipitation process produced BNZ nanoparticles with highly improved solubility compared with the R-BNZ, which may have a direct impact on the absorption and further efficacy of BNZ against *T*. *cruzi*.

As seen in Fig 3, all *Tc*N-infected and treated mice remained viable during those 30 days and 60 days follow-up periods, while only 15% of infected untreated mice survived, confirming the anti-parasitic efficacy of all four formulations investigated. Keeping in mind that the usual dose of BNZ is 100 mg/kg/day [7, 26, 27, 28], it is worth mentioning that BNZ-nps increased the survival rate of infected mice at a much lower dose (10 mg/kg/day). This finding could be related to the increased solubility of BNZ when formulated as nanoparticles, as compared to the raw drug, which may improve the drug absorption and, consequently, higher amounts of drug would be available to produce the desire antiparasitic effect [29].

Moreover, serum samples were analyzed by ELISA and it was found that IgG titers specific to *T*. *cruzi* of all mice were significantly reduced after administration of BNZ-nps in a dose-dependent manner. Treatment with BNZ-nps at 10 mg/kg/day resulted in a 33% decreased IgG titer at 3 months post-infection, and antibodies were reduced to 50% at 6 months. Treatment with 25 mg/kg/day of BNZ-nps resulted in 50% of mice with negative IgG titers at 3 months, and 100% were negative at 6 months. In addition, no specific antibodies to *T*. *cruzi* were found at 3 months post treatment in mice treated with BNZ-nps administered at 50 mg/kg/day, while *T*. *cruzi* antibodies were detected in mice treated with the same dose of R-BNZ. Thus, it should be worth noting that even 10 mg/kg/day of BNZ-nps were sufficient to limit the impact of *T*. *cruzi* infection. Extending the experiment for another few months is likely to result in serologically negative mice, as described for infected human patients [30].

It is known that *T*. *cruzi* infection may be reactivated under immunosuppression, increasing parasitemia as the most important feature of reactivation [31]. Immunosuppressive therapy is a very useful tool to detect residual parasites by PCR after parasiticide treatment in experimental infection [26]. In our work (Fig 5), it was shown that low doses of BNZ-nps (25 mg/kg/day) resulted in 40% negative PCRs in the immunosuppressed mice, indicating the potential importance of nanoformulated BNZ in experimental therapy. Moreover, it is known that Chagas disease leads to heart tissue damage in chronically infected patients [31]. Herein, after a dose-dependent treatment with BNZ-nps, a reduction of the inflammatory cardiomyopathy and the associated fibrosis that occurs in the chronic phase of *T*. *cruzi* infection, was confirmed. It was previously shown the trypanocidal effect of BNZ-nps on intracellular amastigotes in Vero cells and primary cultures of myocytes [12]. In this case, the inhibitory effect was observed with lower BNZ-nps doses than those used for R-BNZ, suggesting that BNZ-nps may enter the cells. As documented in trypanosomiasis infection, the generation of ROS have been found after treatment with BNZ [32]. In this study, the intracellular ROS production was observed after cell incubation with both BNZ-nps and R-BNZ, indicating that nanoformulated BNZ is following the same reductive degradation pathway as R-BNZ and other nitroaromatic compounds, involving type II nitroreductases [33]. In the present study we attempted to demonstrate the significance of nanoformulated BNZ as a novel alternative tool for successful treatment of *T*. *cruzi* infection. Further studies will be needed in order to design a stable and safe BNZ nanoformulation, considering that this novel approach can lead to significant differences in the efficacy, safety, physicochemical properties and pharmacokinetic/pharmacodynamic profile of BNZ in comparison with the conventional and available medicine. In addition, BNZ-nps might lower the frequency of administration while providing a significant enhance of the pharmacological action and reducing the undesirable effects, thus improving the therapeutic compliance of the patients.
